# Optimal caliper widths for propensity-score matching when estimating differences in means and differences in proportions in observational studies

**DOI:** 10.1002/pst.433

**Published:** 2010-04-27

**Authors:** Peter C Austin

**Affiliations:** a Institute for Clinical Evaluative SciencesToronto, Ont., Canada; b Dalla Lana School of Public Health Sciences, University of TorontoOnt., Canada; c Department of Health Management, Policy and Evaluation, University of TorontoOnt., Canada

**Keywords:** propensity score, observational study, binary data, risk difference, propensity-score matching, Monte Carlo simulations, bias, matching

## Abstract

In a study comparing the effects of two treatments, the propensity score is the probability of assignment to one treatment conditional on a subject's measured baseline covariates. Propensity-score matching is increasingly being used to estimate the effects of exposures using observational data. In the most common implementation of propensity-score matching, pairs of treated and untreated subjects are formed whose propensity scores differ by at most a pre-specified amount (the caliper width). There has been a little research into the optimal caliper width. We conducted an extensive series of Monte Carlo simulations to determine the optimal caliper width for estimating differences in means (for continuous outcomes) and risk differences (for binary outcomes). When estimating differences in means or risk differences, we recommend that researchers match on the logit of the propensity score using calipers of width equal to 0.2 of the standard deviation of the logit of the propensity score. When at least some of the covariates were continuous, then either this value, or one close to it, minimized the mean square error of the resultant estimated treatment effect. It also eliminated at least 98% of the bias in the crude estimator, and it resulted in confidence intervals with approximately the correct coverage rates. Furthermore, the empirical type I error rate was approximately correct. When all of the covariates were binary, then the choice of caliper width had a much smaller impact on the performance of estimation of risk differences and differences in means. Copyright © 2010 John Wiley & Sons, Ltd.

## 1. INTRODUCTION

Observational studies are increasingly being used to estimate the effects of treatments and exposures on health outcomes. In randomized controlled trials, randomization ensures that, in expectation, the baseline characteristics of treated subjects do not differ systematically from those of untreated subjects. However, in observational studies, treated subjects often differ systematically from untreated subjects in both measured and unmeasured baseline characteristics. Therefore, statistical methods must be used to adjust for systematic differences between treated and untreated subjects when estimating the effects of treatment on outcomes using observational data.

Propensity-score methods are being used with increasing frequency to account for treatment selection bias when estimating causal treatment effects using observational data. The propensity score is defined to be the probability of exposure to the treatment conditional on a subject's observed baseline characteristics [1,2]. A popular approach to using the propensity score is propensity-score matching [1,3,4]. In propensity-score matching, matched sets of treated and untreated subjects with similar values of the propensity score are formed. The effect of treatment on outcomes is then estimated in the matched sample consisting of all matched sets. A common implementation of propensity-score matching is pair-matching without replacement within a specified caliper distance [5–7]. Using this approach, pairs of treated and untreated subjects are formed such that the difference in propensity scores between matched subjects differs by at most a fixed distance (the caliper width). In matching without replacement, each subject can be included in at most one matched set. In the medical literature, there is no consistency in the calipers that have been used for forming matched sets [5–7]. Intuitively, the choice of caliper should reflect the variance-bias trade-off: using narrower calipers will result in the matching of more similar subjects. This should reduce bias by reducing systematic differences between matched treated and untreated subjects. However, it may also result in a reduction in the number of matched subjects, thereby increasing the variance of the estimated treatment effect. Using wider calipers should have the opposite effect. To date, there is a paucity of research on the optimal caliper width for estimating treatment effects when using propensity-score matching.

The objective of this article was to determine the optimal caliper width for propensity-score matching. The article is structured as follows. In Section 2, we present requisite theory and notation. In Section 3, we describe an extensive series of Monte Carlo simulations to examine the performance of different caliper widths for propensity-score matching when estimating risk differences and differences in means. In Section 4, we present an empirical case study in which we examine the impact of caliper width on estimates of the effect of *β*-blocker therapy on survival using a large sample of patients hospitalized with heart failure. Finally, in Section 5, we summarize our findings.

## 2. THEORY AND NOTATION

Let *X* denote a vector of observed baseline covariates, and let *Y* denote either a continuous or binary outcome variable (in the binary context *Y* = 1 denotes success or the presence of a condition and *Y* = 0 denotes failure or the absence of a condition). Let *Z* denote a binary or dichotomous treatment (*Z* = 1 denoting treated; *Z* = 0 denoting not treated). Then, the propensity score is defined as:
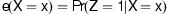
We now briefly describe the potential outcomes framework, using the notation of Imbens [8]. Each subject in the sample has a pair of potential outcomes: *Y*_*i*_(0) and *Y*_*i*_(1), the outcome under the control treatment and the outcome under the active treatment, respectively. However, for each subject, only one of the potential outcomes is observed:
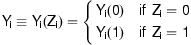
 Two possible treatment effects are the average treatment effect (ATE) and the average treatment effect for the treated (ATT). These are defined as:
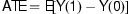
and
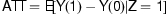
 Although both sample-average and population-average estimates of treatment effect can be defined, we do not make this distinction throughout the article. The ATE is the average effect, at either the population or sample level, of moving the entire population (sample) from untreated to treated. The ATT is the ATE, at either the population or sample level, on the subjects who were ultimately treated.

Imbens notes that propensity-score matching methods allow for the estimation of the ATT, rather than the ATE [8]. The treatment effect is then estimated as the average of the within-pair differences of the outcome. Variance estimation must account for the matched nature of the propensity-score matched sample [8,9].

## 3. MONTE CARLO SIMULATIONS

In this section, we describe and report the results for a series of Monte Carlo simulations used to examine the impact of caliper width on the estimation of the treatment effect. We examined the impact of caliper width on reduction in bias, mean squared error (MSE), coverage of confidence intervals, and type I error rates. We examined two different types of outcomes: dichotomous outcomes and continuous outcomes. Our primary focus was on binary outcomes because they occur more frequently in the medical literature than do continuous outcomes [10]. For binary outcomes, our focus was on estimating risk differences rather than odds ratios for two reasons. First, risk differences are a more natural treatment effect for causal effects in the potential outcomes framework. Second, several clinical commentators have argued that the risk difference (and its reciprocal, the number needed to treat) is more meaningful for clinical decision making than are relative measures of effect, such as relative risks or odds ratios [11–14]. Furthermore, propensity-score matching has been shown to perform poorly for estimating odds ratios [15,16]. Second, we examined scenarios in which the outcome was continuous, and focused on difference in means as the measure of treatment effect. This was to facilitate the comparison of our findings with those of earlier studies whose focus was on estimating difference in means.

### 3.1. Methods

We randomly generated data so that it would be similar to the data considered in the case study in Section 4. In particular, we simulated data so that approximately 25% of the sample was exposed to the treatment. Our simulations were designed to induce a specific ATT, the measure of effect that is estimated when propensity-score matching is used.

#### 3.1.1. Data generation – binary outcomes *(*risk differences*)*

We simulated data such that the probability of the outcome would be approximately 0.29 if all subjects in the population were not exposed (this was the marginal probability of the outcome in the case study examined in Section 4). We then examined scenarios in which the risk differences due to treatment in treated subjects were 0, −0.02, −0.05, −0.10, and −0.15 (i.e. absolute reductions in the probability of the outcome due to treatment were 0, 0.02, 0.05, 0.10, and 0.15). The non-null risk differences are equivalent to NNTs of 50, 20, 10, and 7, respectively. Thus, we generated data so as to induce a specified ATT.

It is difficult to use a conditional data-generating process to generate binary outcomes and exposure such that treatment causes a specific risk difference in the treated subjects. Our data-generating process used the fact that the risk differences are collapsible: the average subject-specific risk difference is equal to the population or marginal risk difference [17]. Our data-generating process has been described in greater detail elsewhere [18], has been used in a similar study [19], and is a modification of a data-generating process for inducing marginal odds ratios of specific magnitudes that has been described elsewhere [20]. We describe our method briefly.

First, we randomly generated 10 independent covariates (*X*_1_–*X*_10_) from independent standard normal distributions for each of 10,000 subjects. We then assumed that the following logistic regression model related the probability of treatment to these 10 baseline covariates:


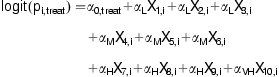
(1)

We then generated a treatment status indicator (*Z*_*i*_) for each subject from a Bernoulli distribution with subject-specific probability equal to ρ_*i,treat*_. Those subjects with *Z*_*i*_ = 1 denoted the treated subjects in whom the ATT is defined. We assumed that the following logistic regression model related the probability of the outcome to these covariates and an indicator variable (*Z*) denoting treatment:


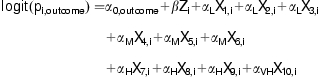
(2)

In the above regression model, *p*_*i*,outcome_ denotes the probability of the outcome for the *i*th subject and *β* denotes the log-odds ratio relating the treatment to the outcome. We then generated subject-specific outcomes from a Bernoulli distribution with probability *p*_*i,*outcome_. The regression coefficients for the baseline covariates in the above two regression models were set as follows: *α*_L_ = log(1.1), *α*_M_ = log(1.25), *α*_H_ = log(1.5), and *α*_VH_ = log(2). These are intended to reflect low, medium, high, and very high effect sizes. We fixed the value of *α*_*0*,outcome_ = log(0.29/0.71) so that the probability of the event occurring in the population if all the subjects were untreated would be approximately 0.29 (to reflect the scenario observed in the case study in Section 4). To induce a risk difference of 0, *β* was set to be 0. For the risk differences of −0.02, −0.05, −0.10, and −0.15, the required value of *β* equaled 0.9077272, 0.7836084, 0.6086645, and 0.4658031, respectively. The reader is referred elsewhere for a more detailed explanation of how these values of *β* were determined [18]. Note that as we are estimating marginal or population-average risk differences, the value of *β* selected will depend on the distribution of baseline covariates in the population. Furthermore, because we are estimating the ATT, the value of *β* will also depend on the population of treated subjects.

#### 3.1.2. Data generation – continuous outcomes

We modified the data-generating process described above to generate a continuous outcome for each subject. We used formula (1) to generate a treatment status for each subject. We then modified formula (2) to generate a continuous outcome for each subject:


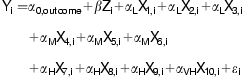
(3)

where 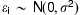. As differences in means are collapsible and there are no constraints on the response for each subject, we used a conditional regression model to generate outcomes for each subject. The regression coefficients for the baseline covariates in the above regression model were set as follows: *α*_L_ = 1.1, *α*_M_ = 1.25, *α*_H_ = 1.5, and *α*_VH_ = 2. These were intended to reflect low, medium, high, and very high sizes. We fixed the value of *α*_*0*,outcome_ = 0. The value of *σ*^2^ was set to 127.6056. This value was selected because it would induce a model *R*^2^ of 0.13, implying that the 10 measured baseline covariates explain 13% of the variation in the outcome. This has been described by Cohen as a medium effect size [21]. We considered five different values of *β* in the outcomes-generating process: 0, 1.1, 1.25, 1.5, and 2. Thus, exposure to the treatment increased the mean of the response variable *Y* by 0, 1.1, 1.25, 1.5, and 2 units, respectively.

The above scenario assumed that the 10 covariates (*X*_1_–*X*_10_) were all independently distributed standard normal random variables. As a sensitivity analysis, we considered four additional scenarios. In the second scenario, the 10 covariates were from a multivariate normal distribution such that the mean and variance of each random variable were equal to 0 and 1, respectively, while the correlation between pairs of random variables was equal to 0.25. In the third scenario, the first five covariates (*X*_1_–*X*_5_) were assumed to be independent Bernoulli random variables with parameter 0.5, while the last five covariates (*X*_6_–*X*_10_) were assumed to be independent standard normal random variables. In the fourth scenario, the first nine covariates were assumed to be independent Bernoulli random variables with parameter 0.5, while the tenth covariate was a standard normal random variable. In the fifth scenario, all 10 covariates (*X*_1_–*X*_10_) were independent Bernoulli random variables with parameter 0.5. The value of *α*_*0*,treat_,*α*_*0*,outcome_, and *β* were modified to preserve the proportion of treated subjects, the marginal probability of the outcome, and the required treatment effect. We refer to the five scenarios as the independent normal covariates scenario, the correlated normal covariates scenario, the first mixed covariates scenario, the second mixed covariates, and the binary covariates scenario, respectively.

### 3.2. Statistical analyses

For each outcome (binary vs continuous) and each magnitude of treatment effect, we randomly generated 1,000 data sets with the required treatment effect (each randomly generated data set consisted of 10,000 subjects as described above). Propensity-score matching was used to construct a matched sample consisting of pairs of treated and untreated subjects with propensity scores that lay within the specified caliper width. We matched subjects on the logit of the propensity score using a caliper of width equal to 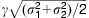, where 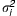 is the variance of the logit of the propensity score in the *i*th group [4]. We allowed *γ* to range from 0.05 to 2.50 in increments of 0.05. Thus, 50 different propensity-score matched samples were formed from each randomly generated data set. The rationale for matching on the logit of the propensity score is that the logit of the propensity score is more likely to be normally distributed than the propensity score itself. Cochran and Rubin determined the reduction in bias when matching on a normally distributed continuous confounding variable using a caliper width that was defined to be a proportion of the standard deviation of that confounding variable [22]. Thus, there is greater rationale for matching on a caliper that is a function of the variance of the propensity score than on a fixed caliper width that is selected independent of the distribution of the propensity score.

#### 3.2.1. Statistical analyses – binary outcomes

Once a propensity-score matched sample had been formed, the absolute risk reduction was estimated as the difference between the proportion of treated subjects experiencing the outcome and the proportion of untreated subjects experiencing the outcome in the matched sample. The statistical significance of the risk difference was tested using McNemar's test for correlated binomial proportions [23], because previous research indicated that accounting for the matched nature of the sample results in superior inference compared with ignoring the matched nature of the sample [9]. Similarly, confidence intervals for the difference in proportions were constructed using methods that account for the matched nature of the sample [23]. Assume that in the matched sample, there are *a* pairs in which both the treated and untreated subjects experienced the event; *b* pairs in which the treated subject experienced the event while the untreated subject does not; *c* pairs in which the untreated subject experienced the event while the treated subject does not; and *d* pairs in which both the treated and untreated subjects did not experience the event. The difference in the probability of the event between treated and untreated subjects is estimated by 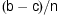, where *n* is the number of matched pairs. The variance of the difference in proportions is estimated by 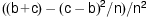
[23]. We also estimated the crude (unadjusted) risk difference in each simulated data set.

For each true risk difference and for a given value of *γ*, we calculated the mean estimated risk difference across the 1,000 simulated data sets. We determined the reduction in bias due to matching on the propensity score. Reduction in bias was defined to be equal to 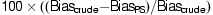, where Bias_crude_ denotes the bias in estimating the treatment effect with the crude or unadjusted estimator in the full or unmatched sample, while Bias_PS_ denotes the bias in estimating the treatment effect when using propensity-score matching. We also calculated the proportion of estimated 95% confidence intervals that contained the true risk difference. We computed the MSE of the estimate. When the true risk difference was 0 (null treatment effect), we estimated the empirical type I error rate as the proportion of simulated data sets in which the null hypothesis that the risk difference was equal to zero was rejected at a 0.05 significance level.

#### 3.2.2. Statistical analyses – continuous outcomes

Within the propensity-score matched sample, let *Y*_T,i_ and *Y*_C,i_ denote the outcome for the treated and untreated subjects in the *i*th matched set, respectively. Then let 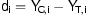 denote the within-matched pair difference in outcome between treated and untreated subject. Then the treatment effect was estimated by 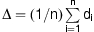, where *n* denotes the number of matched pairs. A one-sample *t*-test was used to test the hypothesis that *Δ* was equal to zero. The standard error of the estimated difference in means was determined, along with a 95% confidence interval for *Δ*. Reduction in bias was determined, as was the MSE of the estimated difference in means.

### 3.3. Results

We report our results for the two different metrics (risk differences vs differences in means) separately.

#### 3.3.1. Results – binary outcomes *(*risk differences*)*

**Figure 1:**
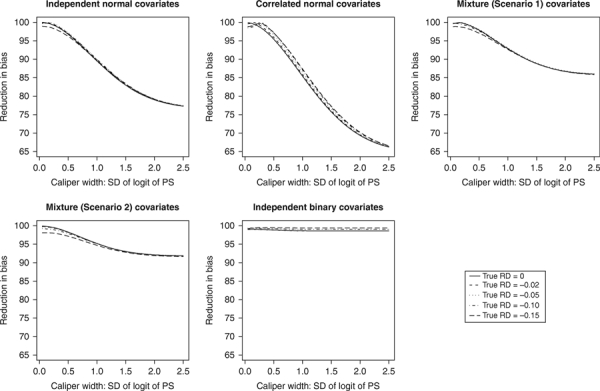
Caliper width and reduction in bias: risk differences.

**Figure 2:**
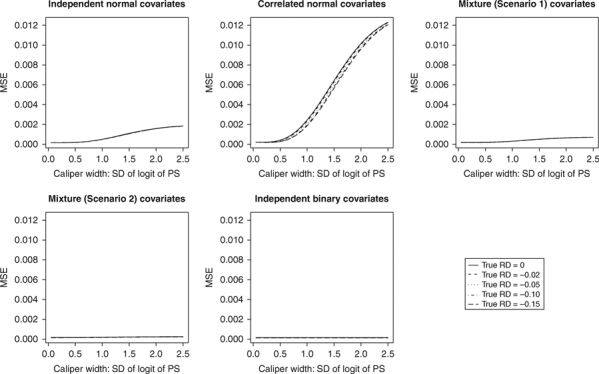
Caliper width and MSE: risk differences.

**Figure 3:**
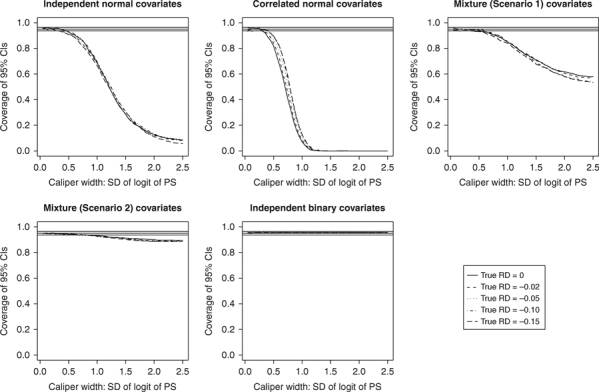
Caliper width and coverage of 95% confidence intervals: risk differences.

**Figure 4:**
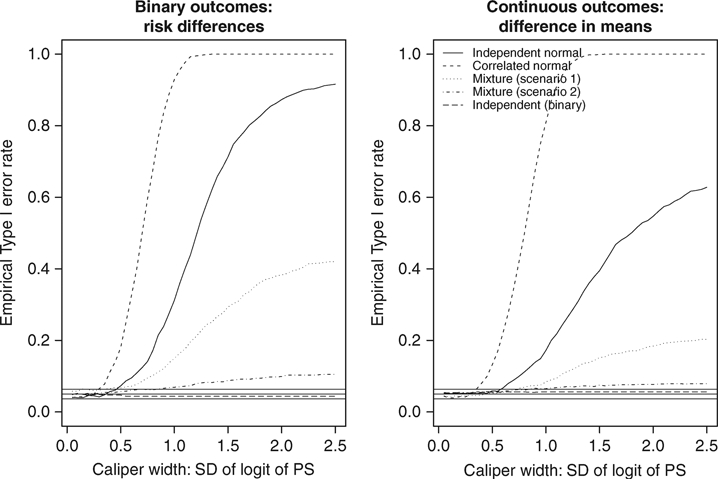
Caliper width and Type 1 error rates.

The relationship between *γ* and the percent reduction in bias, MSE, and empirical coverage rates of 95% confidence intervals are described in Figures 1–3, respectively. Within each figure, there is one panel for each of the five scenarios examined. The relationship between *γ* and type I error is described in the left panel of Figure 4.

The relationship between *γ* and the percentage reduction in bias is described in Figure 1. In the independent standard normal covariates scenario, the use of *γ* equal to 0.05, 0.15, 0.15, 0.05, and 0.05 maximized the reduction in bias when the true risk difference was 0, −0.02, −0.05, −0.10, and −0.15, respectively. For all the five risk differences, the reduction in bias was at least 98.9%, and in four of the cases was at least 99.9%. For the other four covariate scenarios, the value of *γ* that maximized reduction in bias ranged from 0.05 to 0.30, depending on the true risk reduction and the covariate scenario. In comparing the five panels in Figure 1, one observes that the range in the reduction of bias as *γ* varied between 0.05 and 2.50 decreased as the number of continuous covariates decreased and the number of binary covariates increased. When all the covariates were binary, the choice of *γ* had a minimal impact on the reduction in bias.

The relationship between caliper width and the MSE of the estimated risk difference is described in Figure 2 for the five different covariate scenarios (one should note that the same scale is used on the vertical axis of each of the five panels). In the independent standard normal covariates scenario, the use of *γ* equal to 0.20 minimized MSE when the risk difference was equal to 0, −0.02, −0.05, and −0.10. When the risk difference was equal to −0.15, then MSE was minimized when *γ* was equal to 0.10. For the other four scenarios, the value of *γ* that minimized MSE ranged from 0.05 to 0.70. When at least one of the covariates was continuous, the value of *γ* that minimized MSE ranged from 0.05 to 0.30. In comparing the five panels of Figure 2, one notes that the relative differences in the MSE of the estimated risk difference across the range of *γ* decreased as the number of binary covariates increased. When only one of the covariates was normally distributed or when all of the covariates followed a Bernoulli distribution, then the choice of *γ* had a minimal impact on MSE.

The relationship between caliper width and the empirical coverage rates of 95% confidence intervals is described in Figure 3 for the five different covariate scenarios. Given our use of 1,000 simulated data sets, any empirical coverage rate that is less than 0.9365 or that exceeds 0.9635 is statistically significantly different from 0.95 using a statistical test based on the conventional normal approximation to the binomial distribution. Horizontal lines denoting coverage rates of 0.9365, 0.95, and 0.9635 have been added to each panel. The values of *γ* that resulted in empirical coverage rates that were not statistically significantly different from 0.95 varied according to the true risk difference and according to the covariate scenario. However, one notes that using a value of *γ* that was less than or equal to 0.5 tended to result in 95% confidence intervals with approximately correct coverage rates (coverage rates ranged from 0.82 to 0.96, depending on the covariate scenario and the true risk difference). When comparing the different panels of Figure 3, one notes that in the independent normal, correlated normal and the two mixed covariates scenarios, the relationship between *γ* and the empirical coverage rates is relatively flat for values of *γ* between 0.05 and 0.50. However, the empirical coverage rates decreases as *γ* increases beyond this interval. Furthermore, a phenomenon similar to that described above was observed: the relative differences in coverage rates across the range of *γ* were smaller as the number of continuous covariates decreased.

The relationship between caliper width and the empirical type I error rate is described in Figure 4. Owing to the use of 1,000 simulated data sets, any empirical type I error rate that is less than 0.0365 or that exceeds 0.0635 is significantly different from 0.05. Horizontal lines denoting type I error rates of 0.0365, 0.05, and 0.0635 are superimposed on the figure. In the continuous and mixed covariate scenarios, the empirical type I error rate increased with increasing *γ*. When at least one covariate was normally distributed, then selecting *γ* to be at most 0.25 resulted in the empirical type I error rates not being significantly different from 0.05. In the binary covariate scenario, all values of *γ* resulted in approximately correct type I error rates. Finally, one notes that the range of empirical type I error rates decreased as the number of continuous covariates decreased.

#### 3.3.2. Results – continuous outcomes

**Figure 5:**
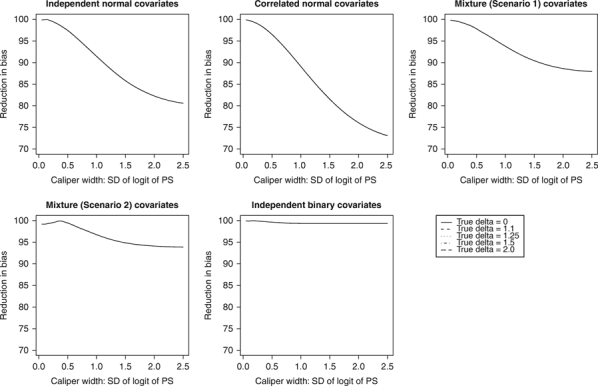
Caliper width and reduction in blas: difference in means.

**Figure 6:**
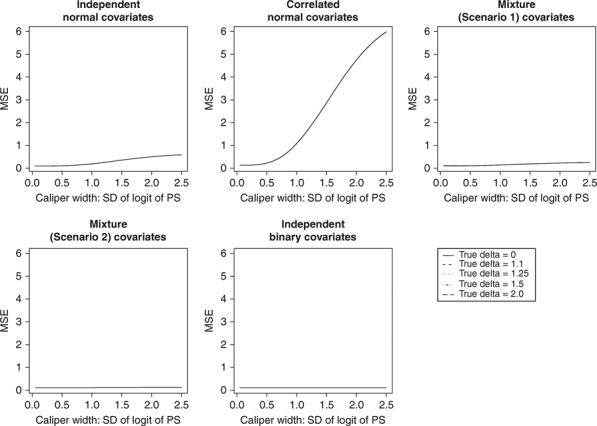
Caliper width and MSE: difference in means.

**Figure 7:**
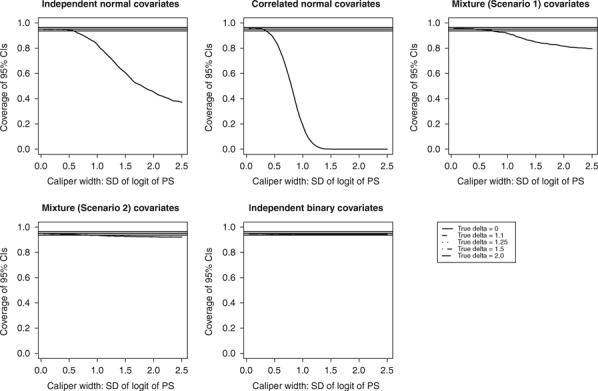
Caliper width and coverage of 95% confidence intervals: difference in means.

The relationship between *γ* and the percent reduction in bias, MSE, and empirical coverage rates of 95% confidence intervals are reported in Figures 5–7, respectively. The relationship between *γ* and type I error is described in the right panel of Figure 4.

The relationship between *γ* and the percentage reduction in bias is described in Figure 5. In the independent normal covariates scenario, the use of *γ* equal to 0.15 maximized the reduction in bias, regardless of the true difference in means. For all the five true differences in means, the reduction in bias was equal to 99.9%. The value of *γ* that maximized bias reduction varied from 0.05 to 0.35 across the other four covariate scenarios. In all the five covariate scenarios, the reduction in bias was at least 99.8% when *γ* was equal to the value that maximized bias reduction. In Figure 5, one observes a similar phenomenon as in the case with the risk differences and binary outcomes: the range in the percent reduction in bias across the range of *γ* decreased as the number of continuous covariates decreased.

The relationship between caliper width and the MSE of the estimated difference in means is described in Figure 6 for the five different covariate scenarios. In the independent normal covariates scenario, the use of *γ* equal to 0.20 minimized MSE, regardless of the true difference in means. In the correlated normal covariates scenario, the use of *γ* equal to 0.1 minimized MSE. In the first mixed covariates scenario, the use of *γ* equal to 0.35 minimized MSE, while in the second mixed covariates scenario, the use of *γ* equal to 0.55 minimized MSE, regardless of the true risk difference. In the binary covariate scenario, the use of *γ* equal to 0.8 minimized MSE, regardless of the true difference in means. In examining Figure 6, one observes a similar phenomenon as with the risk differences and binary outcomes: the range in MSE across the spectrum of *γ* decreased as the number of continuous covariates decreased. When there were either one or no continuous covariates, the choice of *γ* had a negligible impact on the MSE.

The relationship between caliper width and the empirical coverage rates of 95% confidence intervals is described in Figure 7 for the five different covariate scenarios. The values of *γ* that resulted in empirical coverage rates that were not statistically significantly different from 0.95 varied according to the covariate scenario. However, across all covariate scenarios, the values of *γ* that were at most 0.35 resulted in 95% confidence intervals with approximately the correct coverage rates, regardless of the true difference in means. When at most one of the covariates was normally distributed, the choice of *γ* had minimal impact on the empirical coverage rates of the 95% confidence intervals. In comparing the different panels of Figure 7, one observes a similar phenomenon as with the risk differences and binary outcomes: the range in empirical coverage rates of 95% confidence intervals across the spectrum of *γ* decreased as the number of continuous covariates decreased.

The relationship between caliper width and the empirical type I error rate is described in the right panel of Figure 4. In the two continuous covariate scenarios and the two mixed covariate scenarios, the empirical type I error rate increased with increasing *γ*. In the independent normal covariates scenario, the values of *γ* between 0.05 and 0.55 resulted in type I error rates that were approximately correct; when the covariates were correlated normal random variables, then the values of *γ* between 0.05 and 0.35 resulted in type I error rates that were approximately correct. In the first mixed covariate scenario, selecting values of *γ* between 0.05 and 0.70 resulted in empirical type I error rates that were not significantly different from the advertised rate. In the second mixed covariate scenario, selecting values of *γ* between 0.05 and 0.80 resulted in empirical type I error rates that were not significantly different from the advertised rate. In the binary covariate scenario, all the values of *γ* resulted in approximately correct type I error rates. Finally, as with the risk differences for binary outcomes, one observes that the range of the empirical type I error rates across the spectrum of *γ* decreased as the number of continuous covariates decreased.

## 4. CASE STUDY

### 4.1. Data sources

Detailed clinical data were obtained by retrospective chart review on a sample of 7,613 patients discharged alive with a diagnosis of heart failure between 1 April 1999 and 31 March 31 2001 from 103 acute care hospitals in Ontario, Canada. Further details of the data obtained were provided elsewhere [24]. These data were collected as part of the Enhanced Feedback for Effective Cardiac Treatment (EFFECT) Study, an ongoing initiative intended to improve the quality of care for patients with cardiovascular disease in Ontario [25]. Data on patient demographics, vital signs at presentation, results of physical examination at presentation, medical history, and results of laboratory tests were collected for this sample. Subjects with missing data on key continuous baseline covariates were excluded from this study. In this study, we examined receipt of a prescription for a *β*-blocker at discharge as the exposure of interest. The demographic and clinical characteristics of the treated and untreated subjects are described in Table I. Continuous and categorical variables were compared between treated and untreated subjects using the Wilcoxon Rank Sum test and the χ^2^ test, respectively. Standardized differences are also reported for comparing the mean of variables between the treatment groups [26]. Systematic differences in several variables, including age, systolic blood pressure, heart rate, history of previous myocardial infarction, history of chronic obstructive pulmonary disease, and dementia, were observed between the treatment groups. Overall, 27.3% of patients received a prescription for a *β*-blocker at discharge. The outcome of interest was death within 1 year of hospital discharge. A total of 27.7% of the subjects died within 1 year of hospital discharge.

**Table I:** Baseline characteristics of *β*-blocker and non-*β*-blocker patients in the case study

	Median (25th percentile–75th percentile) or *N* (%)			
			
Baseline characteristics	*β*-blocker: No (*N* = 5535)	*β*-blocker: Yes (*N* = 2078)	Standardized difference	*P*-value
*Demographic characteristics*				
Age (years)	78 (70–84)	75 (67–82)	0.24	<0.001
Female	2809 (50.7%)	1011 (48.7%)	0.04	0.103
*Vital signs on admission*				
Systolic blood pressure, mmHg	147 (127–170)	150 (130–176)	0.13	<0.001
Heart rate, beats per minute	94 (78–111)	88 (73–108)	0.14	<0.001
Respiratory rate, breaths per minute	24 (20–30)	24 (20–28)	0.09	<0.001
*Presenting symptoms and physical exam*				
Neck vein distension	3002 (54.2%)	1200 (57.7%)	0.07	0.006
S3	518 (9.4%)	232 (11.2%)	0.06	0.018
S4	204 (3.7%)	89 (4.3%)	0.03	0.227
Rales > 50% of lung field	560 (10.1%)	231 (11.1%)	0.03	0.203
*Findings on chest X-ray*				
Pulmonary edema	2772 (50.1%)	1137 (54.7%)	0.09	<0.001
Cardiomegaly	2026 (36.6%)	711 (34.2%)	0.05	0.053
*Past medical history*				
Diabetes	1871 (33.8%)	804 (38.7%)	0.1	<0.001
CVA/TIA	880 (15.9%)	340 (16.4%)	0.01	0.624
Previous MI	1815 (32.8%)	989 (47.6%)	0.31	<0.001
Atrial fibrillation	1675 (30.3%)	530 (25.5%)	0.1	<0.001
Peripheral vascular disease	684 (12.4%)	302 (14.5%)	0.06	0.012
Chronic obstructive pulmonary disease	1074 (19.4%)	191 (9.2%)	0.28	<0.001
Dementia	422 (7.6%)	91 (4.4%)	0.13	<0.001
Cirrhosis	48 (0.9%)	6 (0.3%)	0.07	0.007
Cancer	659 (11.9%)	195 (9.4%)	0.08	0.002
*Electrocardiogram – First available within 48 h*				
Left bundle branch block	834 (15.1%)	293 (14.1%)	0.03	0.29
*Laboratory tests*				
Hemoglobin, g/L	124 (110–138)	125 (111–139)	0.05	0.146
White blood count, 10E9/L	9 (7–12)	9 (7–11)	0.02	0.261
Sodium, mmol/L	139 (136–141)	139 (137–141)	0.08	0.001
Potassium, mmol/L	4 (4–5)	4 (4–5)	0.03	0.12
Glucose, mmol/L	7 (6–11)	8 (6–12)	0.09	<0.001
Blood urea nitrogen, mmol/L	8 (6–12)	8 (6–12)	0	0.522
Creatinine, *μ*mol/L	104 (82–142)	107 (85–144)	0.08	0.002

### 4.2. Statistical analyses

An indicator variable denoting receipt of a *β*-blocker prescription at hospital discharge was regressed on the 28 baseline characteristics described in Table I using a logistic regression model. The estimated propensity score was the predicted probability of receiving a *β*-blocker prescription that was derived from the fitted logistic regression model. Continuous variables were not categorized, and were assumed to have a linear relationship with the log-odds of *β*-blocker use.

Treated and untreated subjects were matched on the logit of the estimated propensity score using calipers of width equal to *γ* of the standard deviation of the logit of the propensity score. We allowed *γ* to range from 0.05 to 2.50 in increments of 0.05. We estimated the absolute reduction in mortality due to *β*-blocker exposure at discharge, the standard error of the estimated risk difference, and the associated 95% confidence interval using methods described in Section 3.2.1.

### 4.3. Results

**Figure 8:**
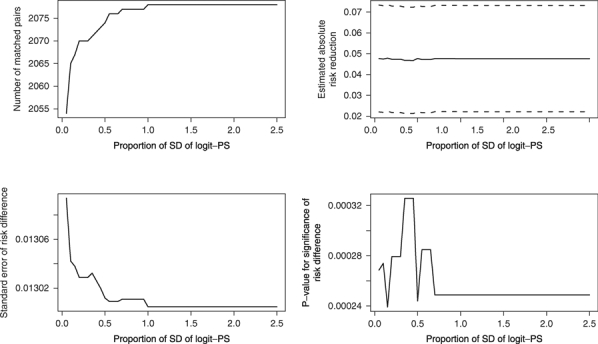
Relationship between caliper width and estimated treatment effect in case study.

The results of the case study are described graphically in Figure 8. The relationship between *γ* and the number of matched pairs is described in the upper left panel of Figure 8. One observes that initially, as *γ* increased, the number of matched pairs increased. However, the choice of *γ* had a small impact on the number of matched pairs. When *γ* was equal to 0.05, 2054 matched pairs were formed. Once *γ* exceeded 0.95, the number of matched pairs remains constant at 2078.

The relationship between *γ* and the estimated risk difference and its associated 95% confidence interval are described in the upper right panel of Figure 8. One observes that the choice of *γ* had a minimal impact on the estimated risk difference and its associated 95% confidence interval. The estimated risk differences ranged from 0.047 to 0.048.

The relationship between *γ* and the standard error of the estimated risk difference is described in the lower left panel of Figure 8. One notes that initially, the estimated standard error of the risk difference decreases with increasing *γ*. However, once *γ* exceeded 0.95, the standard error remains constant.

Finally, the relationship between *γ* and the statistical significance level of the estimated risk difference is described in the lower right panel of Figure 8. One observes that the choice of *γ* had an inconsistent relationship on the statistical significance of the risk difference. However, for all the values of *γ*, the estimated risk difference was statistically significantly different from zero (*P*<0.0004).

## 5. DISCUSSION

We used Monte Carlo simulations to examine the relationship between the caliper width used for propensity-score matching and the performance of estimation of the risk differences and differences in means. We begin by briefly synthesizing our findings.

When estimating differences in means, we found that MSE was minimized by using calipers that were equal to a width of between 0.20 and 0.55 times the standard deviation of the logit of the propensity score when at least one of the covariates were continuous. Furthermore, the use of calipers of these widths tended to result in confidence intervals with approximately correct coverage rates and significance tests with approximately correct empirical type I error rates. When all of the covariates were binary, then MSE was minimized when the calipers had a width equal to 0.8 times the standard deviation of the logit of the propensity score. However, it should be noted that the choice of caliper width had a negligible impact on the performance of estimation when all of the covariates were binary.

When estimating the risk differences, we found that MSE was minimized by using calipers that were equal to a width of between 0.05 and 0.30 times the standard deviation of the logit of the propensity score when at least one of the covariates was continuous. Furthermore, the use of calipers of these widths tended to result in confidence intervals with approximately correct coverage rates and significance tests with approximately correct empirical type I error rates. When all of the covariates were binary, we found that wider calipers had to be used, and that MSE was minimized when the calipers had a width equal to 0.3 to 0.7 times the standard deviation of the logit of the propensity score. As in the case of estimating differences in means, it should be noted that the choice of caliper width had a negligible impact on the performance of estimation when all of the covariates were binary.

In our case study using a large sample of patients hospitalized with heart failure, we observed that the different choices for *γ* resulted in qualitatively similar estimates of the absolute reduction in the probability of mortality within 1 year due to receipt of a *β*-blocker prescription at hospital discharge. Similarly, the choice of *γ* had a minimal impact on the statistical significance of the estimated risk difference.

Recent reviews of propensity-score matching in the medical literature have documented that a wide choice of calipers have been used in applied applications [5–7]. In most cases, the choice of caliper appeared to have been *ad hoc*, and not based on substantive theory. Indeed, there is a paucity of research into the relative performance of different calipers for propensity-score matching. Cochran and Rubin examined matching in the setting in which a continuous response variable was linearly related to both a dichotomous exposure and to a single continuous confounding variable [22]. They examined the reduction in bias when matching on the continuous confounding variable using calipers of width equal to 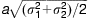, where 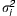
is the variance of the continuous confounding variable in the *i*th group. When 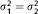, they found that using values of *a* equal to 0.2, 0.4, 0.6, 0.8, and 1.0 eliminated 99%, 95%, 89%, 82%, and 74% of the bias due to the confounding variable, respectively. In a subsequent article, Rosenbaum and Rubin examined the construction of a control group using matching on the propensity score [4]. They examined matching on the logit of the propensity score using calipers that were equal to 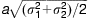, where 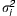 is the variance of the logit of the propensity score in the *i*th group. Rosenbaum and Rubin suggest that matching on the logit of the propensity score using a given value of *a* will remove the same degree of bias as will matching on a single continuous confounding variable using the same value of *a*. Thus, if the variance of the logit of the propensity score was the same in both groups, using calipers of width equal to 0.2 of the pooled standard deviation of the logit of the propensity score would eliminate approximately 99% of the bias due to measured confounding variables, while using calipers of width equal to 0.6 of the pooled standard deviation of the logit of the propensity score would eliminate approximately 89% of the bias due to measured confounding variables. The first result is similar to our finding that, when estimating differences in means, using calipers of width equal to 0.2 of the pooled standard deviation of the logit of the propensity score eliminated at least 99.3% of the bias in the crude estimator. The second finding contrasts with our findings, in that we found that using calipers of width equal to 0.6 of the pooled standard deviation of the logit of the propensity score eliminated between 95.2% and 99.6% of the bias in the crude estimator, with the amount of bias reduction dependent on the covariate scenario. Apart from these two articles, there is a dearth of articles that provide guidance on the selection of calipers for use with propensity-score matching. In a recent article, Austin compared the performance of eight different methods for propensity-score matching. Two methods were based on matching on the logit of the propensity score (using calipers of width equal to either 0.2 or 0.6 of the standard deviation of the logit of the propensity score, one method based on 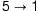 digit matching, and five methods based on fixed caliper widths on the propensity-score scale (0.005, 0.01, 0.02, 0.03, and 0.10) [27]. These methods were selected because they were the ones most frequently used in practice in the medical literature. Matching on the logit of the propensity score using calipers of width equal to 0.2 of the standard deviation of the logit of the propensity score and calipers of width equal to 0.02 or 0.03 tended to have superior performance for estimating treatment effects. However, apart from these studies, there is limited information on how to select the appropriate caliper for use with propensity-score matching.

Both Cochran and Rubin [22] and Rosenbaum and Rubin [4] focused on the impact of caliper width on reduction in bias. In this study, we have focused on reduction in bias, MSE, coverage of confidence intervals, and type I error rates. MSE allows researchers to quantify the trade-off between variance and bias. As suggested in the Introduction, the choice of caliper width reflects an implicit trade-off between variance and bias. Our focus on MSE allows researchers to select a caliper width that optimizes this implicity trade-off. Furthermore, our examination of type I error rates allows researchers to select a caliper width that will result in statistical tests with approximately correct rejection rates.

There are certain limitations to the current study. First, our Monte Carlo simulations were limited to 1000 replications per scenario. The implications of our use of 1000 iterations per scenario was described above in terms of the ability to detect coverage rates and type I error rates that were significantly different from 0.95 and 0.05, respectively. However, more precise results could be obtained with a larger number of iterations. The number of iterations was restricted to 1000 for computational reasons. In the independent normal covariate scenario, the use of 1,000 iterations required approximately 60 days of CPU time on a unix server. Within each simulated data set, 50 propensity-score matched samples were formed. Most of the computer time involved forming the propensity-score matched samples. Given that we examined five different scenarios, the use of additional iterations was not feasible. For similar reasons, we were unable to examine the robustness of our findings under a wider range of scenarios. A second limitation was that we focused only on the impact of caliper width on estimation. We did not focus on other issues such the relationship between caliper width and balance of measured baseline covariates between treated and untreated subjects. An overview of balance diagnostics for use with propensity-score matching is provided elsewhere [28], as is a comparison of the relative ability of the different propensity-score methods to balance measured covariates [29].

We now summarize recommendations for propensity-score matching based on the current study. When estimating differences in means or risk differences, we recommend that researchers match on the logit of the propensity score using calipers of width equal to 0.2 of the standard deviation of the logit of the propensity score. When at least some of the covariates were continuous, then either this value or one close to it minimized the MSE of the resultant estimated treatment effect. It also eliminated at least 98% of the bias in the crude estimator and resulted in confidence intervals with approximately the correct coverage rates. Furthermore, the type I error rate was approximately correct. When all of the covariates were binary, then the choice of caliper width had a much smaller impact on the performance of estimation of the risk differences and differences in means.
